# Chi-square test under indeterminacy: an application using pulse count data

**DOI:** 10.1186/s12874-021-01400-z

**Published:** 2021-09-30

**Authors:** Muhammad Aslam

**Affiliations:** Department of Statistics, Faculty of Science, King Abdulaziz University, Jeddah, 21551 Saudi Arabia

**Keywords:** Chi-square test, Count data, Pulse count, Classical statistics, Uncertainty

## Abstract

**Background:**

The data obtained from the counting process is known as the count data. In practice, the counting can be done at the same time or the time of the count is not the same. To test either the K counts are differed significantly or not, the Chi-square test for K counts is applied.

**Results:**

The paper presents the Chi-square tests for K counts under neutrosophic statistics. The test statistic of the proposed test when K counts are recorded at the same time and different time are proposed. The testing procedure of the proposed test is explained with the help of pulse count data.

**Conclusions:**

From the analysis of pulse count data, it can be concluded that the proposed test suggests the cardiologists use different treatment methods on patients. In addition, the proposed test gives more information than the traditional test under uncertainty.

## Background

The data obtained from the counting process is known as the count data. In practice, the counting can be done at the same time or the time of the count is not the same. To test either the K counts are differed significantly or not, the Chi-square test for K counts is applied. This test is applied to test the null hypothesis either the same training or methods should be applied on K counts against the alternative hypothesis that different training or methods should be applied on K counts. The Chi-square test for K counts under classical statistics is applied under the assumption that K counts are obtained under comparable conditions, see [1, 2]. Worked on the test for testing two means of Poisson distribution [3–10] presented applications of test for count data in a variety of fields.

According to [11], “statistical data are frequently not precise numbers but more or less non-precise also called fuzzy. Measurements of continuous variables are always fuzzy to a certain degree”. Similarly, the counting data is not always exact but may be in intervals or unclear. For example, the weather record data and pulse count data are expressed in intervals than the exact values. In these situations, the existing Chi-square test for K counts under classical statistics may mislead the decision-makers. The fuzzy-based tests may be an alternative to being applied when the count data is intervals. The applications of statistical tests under fuzzy logic can be seen in [12–19].

The fuzzy logic is a special case of neutrosophic logic [20, 21] showed the efficiency of the neutrosophic logic over the fuzzy logic and analysis based on the interval approach. For the application of neutrosophic logic, the reader may refer to [22–26]. The neutrosophic statistics was proposed using the idea of neutrosophic logic by [27]. This is a branch of mathematical statistics that provides the presentation, analysis, and inference of neutrosophic, fuzzy, interval, and indeterminate data. Classical statistics was considered a special case of neutrosophic statistics [28–33] discussed various applications of neutrosophic statistics.

The existing Chi-square test for K counts under classical statistics cannot be applied when the counts are in intervals. In this paper, the design of the Chi-square test for K counts under neutrosophic statistics will be given. We will extend the statistic for counts at the same time and at different times under neutrosophic statistics. The testing of the hypothesis will be given. The application of the proposed test will be given using the pulse counts data. By proposing the test, it is expected that the proposed test will be effective, flexible, informative, and adequate to be applied under uncertainty.

## Results

The pulse rate is theoretically is considered a discrete variable. The application of the proposed test is given using the counts of the pulse rate of 11 patients. The first 11 values of data are obtained from [34] and the next values are generated by simulation. The data is shown in Table 1. A cardiologist is interested to see either the same treatment should be applied to all patients or not. Therefore, the null hypothesis for this case is that the same treatment method should be applied vs. the alternative hypothesis that different methods of treatment should be applied to all patients. As the pulse counts are noted in the same period of time, therefore, the statistic ${\chi}_{1N}^2\epsilon \left[{\chi}_{1L}^2,{\chi}_{1U}^2\right]$ is given in Eq. (1) a suitable statistic to apply for testing the given hypothesis. The neutrosophic form of ${\overline{N}}_{iN}\epsilon \left[{\overline{N}}_{iL},{\overline{N}}_{iU}\right]$ using the given data is given as ${\overline{N}}_{iN}=73.54+91.8{I}_{i\overline{N}};{I}_{i\overline{N}}\epsilon \left[\mathrm{0,0.1989}\right]$.The test statistic ${\chi}_{1N}^2\epsilon \left[{\chi}_{1L}^2,{\chi}_{1U}^2\right]$ for the given data is computed as ${\chi}_{1N}^2=\sum_{i=1}^K\frac{{\left({N}_{iN}-{\overline{N}}_{iN}\right)}^2}{{\overline{N}}_{iN}}=\left[\mathrm{146.97,121.78}\right]$.The neutrosophic form of the statistic ${\chi}_{1N}^2\epsilon \left[{\chi}_{1L}^2,{\chi}_{1U}^2\right]$ using the data is given as ${\chi}_{1N}^2=146.97-121.78{I}_{\chi_{1N}^2};{I}_{\chi_{1N}^2}\epsilon \left[\mathrm{0,0.2068}\right]$. The proposed test will be implemented as follows

- **Step-1:** State the null *H*_0_: all patients should be treated by the same method and alternative hypothesis *H*_1_: patients should be treated with different methods.
- **Step-2:** Let *α* = 5% and critical values are 34.76 and 67.5.
- **Step-3:** Reject *H*_0_ as the values of ${\chi}_{1N}^2\epsilon \left[{\chi}_{1L}^2,{\chi}_{1U}^2\right]$ fall in the rejection region.
- From the study, it can be concluded that cardiologists should use different methods of treatment for the patients.

**Table 1:** The pulse counts data

Patient#			Patient#	*N*_*iN*_	$\frac{{\left({N}_{iN}-{\overline{N}}_{iN}\right)}^2}{{\overline{N}}_{iN}}$
	*N*_*iN*_	$\frac{{\left({N}_{iN}-{\overline{N}}_{iN}\right)}^2}{{\overline{N}}_{iN}}$			
1	[44,68]	[11.87,6.17]	26	[67, 78]	[0.58, 2.07]
2	[62,72]	[1.81,4.27]	27	[76, 97]	[0.08, 0.29]
3	[56,90]	[4.18, 0.04]	28	[95, 117]	[6.26, 6.92]
4	[70,112]	[0.17, 4.44]	29	[86, 99]	[2.11, 0.56]
5	[54,72]	[5.19, 4.27]	30	[66, 100]	[0.77, 0.73]
6	[70,100]	[0.17, 0.73]	31	[77, 88]	[0.16, 0.16]
7	[63,75]	[1.51, 3.07]	32	[88, 99]	[2.84, 0.56]
8	[72,100]	[0.03, 0.73]	33	[83, 112]	[1.22, 4.44]
9	[76,98]	[0.08, 0.42]	34	[56, 65]	[4.18, 7.82]
10	[86,96]	[2.11, 0.19]	35	[45, 78]	[11.08, 2.07]
11	[86,100]	[2.11, 0.73]	36	[87, 97]	[2.46, 0.29]
12	[67, 88]	[0.58, 0.16]	37	[67, 89]	[0.58, 0.09]
13	[89, 100]	[3.25, 0.73]	38	[84, 102]	[1.49, 1.13]
14	[56, 65]	[4.18, 7.82]	39	[90, 109]	[3.68, 3.22]
15	[87, 93]	[2.46, 0.02]	40	[91, 108]	[4.15, 2.86
16	[43, 70]	[12.68, 5.18]	41	[56, 77]	[4.18,2.39]
17	[86, 112]	[2.11, 4.44]	42	[77, 99]	[0.16,0.56]
18	[65, 77]	[0.99, 2.39]	43	[73, 89]	[0.00,0.09]
19	[89, 99]	[3.25, 0.56]	44	[89, 114]	[3.25,5.37]
20	[90, 101]	[3.68, 0.92]	45	[64, 70]	[1.24, 5.18]
21	[77, 89]	[0.16, 0.09]	46	[56, 74]	[4.18, 3.45]
22	[98, 115]	[8.14, 5.86]	47	[91, 117]	[4.15, 6.92]
23	[54, 66]	[5.19, 7.25]	48	[67, 88]	[0.58, 0.16]
24	[75, 89]	[0.03, 0.09]	49	[54, 77]	[5.19, 2.39]
25	[85, 100]	[1.79, 0.73]	50	[92, 100]	[4.63, 0.73]
			**Sum**		[146.9733,121.7865]

## Discussion

**Fig. 1:**
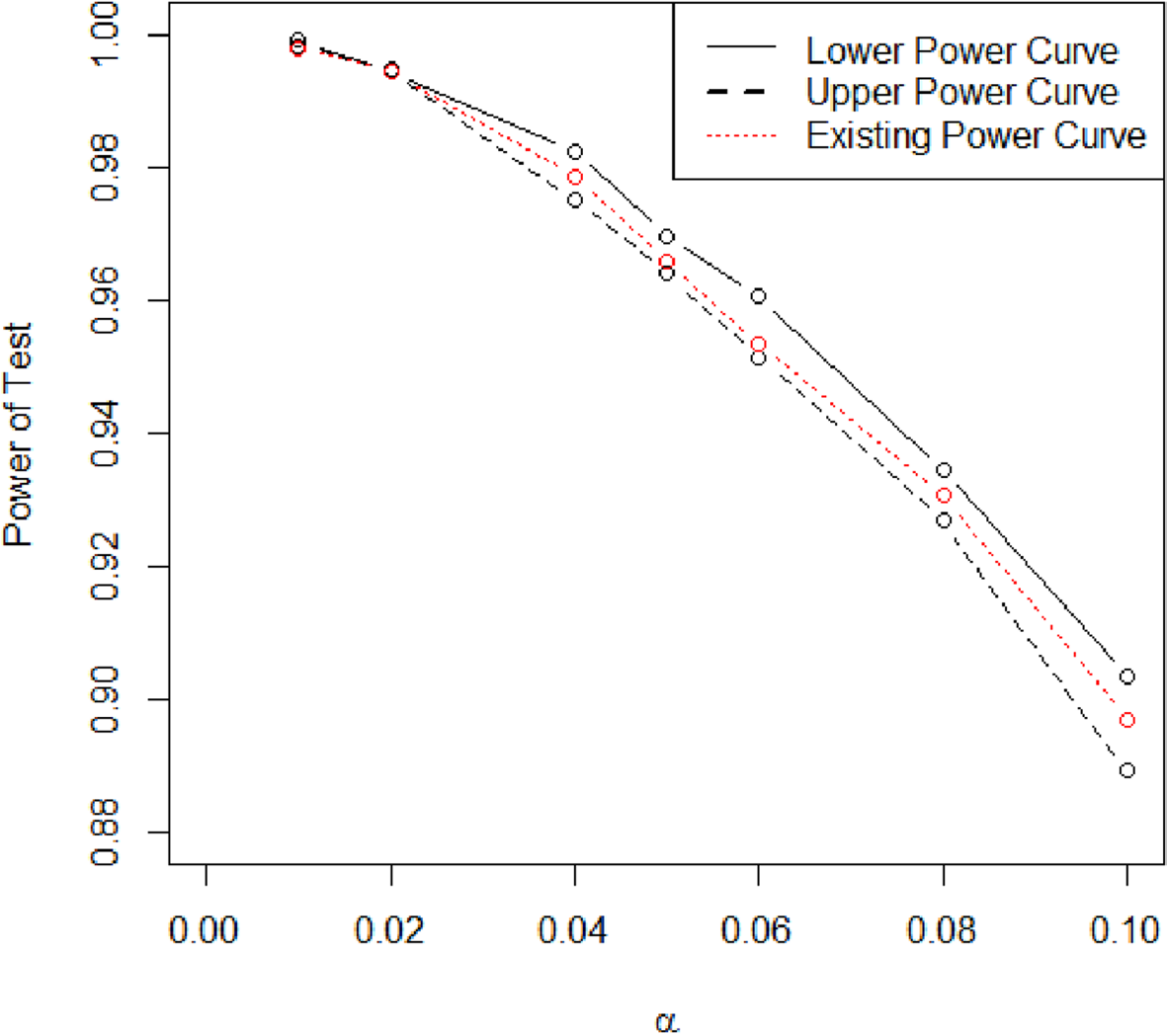
Power curves of the proposed and existing tests at various values of *α*

As mentioned earlier, the neutrosophic form of the proposed test consists of two parts. The first and the second parts presented classical statistics and indeterminate, respectively. The neutrosophic form of ${\chi}_{1N}^2={\chi}_{1L}^2+{\chi}_{1U}^2{I}_{\chi_{1N}^2};{I}_{\chi_{1N}^2}\epsilon \left[{I}_{\chi_{1L}^2},{I}_{\chi_{1U}^2}\right]$ reduces to statistic under classical statistics when ${I}_{\chi_{1L}^2}=0$. The efficiency of the proposed test will be compared with the existing test in terms of uncertainty measures. The neutrosophic form of ${\chi}_{1N}^2\epsilon \left[{\chi}_{1L}^2,{\chi}_{1U}^2\right]$ of pulse count data is ${\chi}_{1N}^2=146.97-121.78{I}_{\chi_{1N}^2};{I}_{\chi_{1N}^2}\epsilon \left[\mathrm{0,0.2068}\right]$. When ${I}_{\chi_{1L}^2}=0$, the value 146.97 presents the existing test statistic. The part $121.78{I}_{\chi_{1N}^2}$ is an indeterminate part and ${I}_{\chi_{1U}^2}$ = 0.2068 is the uncertainty measure associated with statistic ${\chi}_{1N}^2\epsilon \left[{\chi}_{1L}^2,{\chi}_{1U}^2\right]$. From the neutrosophic form, it can be seen that the proposed test statistic can be expressed in interval rather than the exact value. Under uncertainty, the value of the test statistic is from 146.97 to 121.78. From this study, it can be seen that the proposed test gives the results in the indeterminate interval that is expecting under uncertainty. On the other, the proposed statistic gives information about indeterminacy. Under an indeterminate environment, the proposed test has the interpretation like: when *α* = 5%, the probability of committing a type-I error is 0.05, the probability of accepting *H*_0_ is 0.95 and the chance of indeterminacy about the accepting or rejecting *H*_0_ is 0.2068. Let *β* is the probability of rejecting *H*_0_ when it is true. To study the power of test (1 − *β*) for the proposed test and the existing test, various values of the level of significance *α* are considered. The neutrosophic data is generated from 45 to 55 and the values of ${\chi}_{1N}^2\epsilon \left[{\chi}_{1L}^2,{\chi}_{1U}^2\right]$ are computed and compared with the critical values at various levels of *α*. The probability of rejecting *H*_0_ when it is true (*β*) is calculated and used to calculate the power of the test (1 − *β*). The values of (1 − *β*) at various values of *α* are shown in Table 2 and plotted in Fig. 1. From Table 1, it can be seen that as the value of *α* increases from 0.1 to 0.99, the power of the test also increases. Figure 1 clearly indicates that the power curve of the proposed test is higher than the power curve of the existing test. The comparative study shows that the proposed test is efficient, revealing, and stretchy than the existing test. In addition, the proposed test provides higher values of power of the test as compared to the existing test.

**Table 2:** The values of the power of the test for the proposed test

*α*	(1 − *β*)
0.1	[0.9032,0.8891]
0.92	[0.9344,0.9268]
0.94	[0.9606,0.9515]
0.95	[0.9697,0.964]
0.96	[0.9826,0.9751]
0.98	[0.9945,0.9948]
0.99	[0.9992,0.9983]

## Methods

The existing test for K counts under classical statistics is applied under the assumption that the data of counts must be noted under comparable conditions. The existing test to investigate the existing test between K counts can be applied only when the counts are exact, precise, and clear. In this section, the proposed test for K counts will be introduced when the count data is in indeterminate intervals, unclear and vagueness. The proposed test will be designed when the time of counts is equal and not equal. The method of the proposed test when times of counts are equal is designed first. Let *N*_*iN*_ = *N*_*iL*_ + *N*_*iU*_*I*_*iN*_; *I*_*iN*_*ϵ*[*I*_*iL*_, *I*_*iU*_] be neutrosophic counts at *ith* time, where *N*_*iL*_ presents exact counts, *N*_*iU*_*I*_*iN*_ presents inexact or indeterminate counts and *I*_*iN*_*ϵ*[*I*_*iL*_, *I*_*iU*_] be a measure of indeterminacy associated with the counts. Suppose that ${\overline{N}}_{iN}={\overline{N}}_{iL}+{\overline{N}}_{iU}{I}_{i\overline{N}};{I}_{i\overline{N}}\epsilon \left[{I}_{i\overline{L}},{I}_{i\overline{U}}\right]$ be a neutrosophic average of K counts, where ${\overline{N}}_{iL}$ and ${\overline{N}}_{iU}{I}_{i\overline{N}}$ are the determined and indeterminate part of neutrosophic average and ${I}_{i\overline{N}}\epsilon \left[{I}_{i\overline{L}},{I}_{i\overline{U}}\right]$ be the measure of indeterminacy associated with the neutrosophic average. The proposed test will be applied for testing the null hypothesis *H*_0_ : *N*_*iN*_= constant, when *i* = 1, 2, 3, …, *K*. The test statistic under neutrosophic statistics say ${\chi}_{1N}^2\epsilon \left[{\chi}_{1L}^2,{\chi}_{1U}^2\right]$ can be written as follows.

1 $${\chi}_{1N}^2=\sum_{i=1}^K\frac{{\left({N}_{iN}-{\overline{N}}_{iN}\right)}^2}{{\overline{N}}_{iN}};{\chi}_{1N}^2\epsilon \left[{\chi}_{1L}^2,{\chi}_{1U}^2\right],{\overline{N}}_{iN}\epsilon \left[{\overline{N}}_{iL},{\overline{N}}_{iU}\right]$$

The neutrosophic form of the proposed test ${\chi}_{1N}^2\epsilon \left[{\chi}_{1L}^2,{\chi}_{1U}^2\right]$ can be written as.

2 $${\chi}_{1N}^2={\chi}_{1L}^2+{\chi}_{1U}^2{I}_{\chi_{1N}^2};{I}_{\chi_{1N}^2}\epsilon \left[{I}_{\chi_{1L}^2},{I}_{\chi_{1U}^2}\right]$$

Note that the proposed statistic ${\chi}_{1N}^2\epsilon \left[{\chi}_{1L}^2,{\chi}_{1U}^2\right]$ is the extension of the test statistic under classical statistics. The proposed test statistic ${\chi}_{1N}^2\epsilon \left[{\chi}_{1L}^2,{\chi}_{1U}^2\right]$ reduces to classical statistic ${\chi}_{1L}^2$ when ${I}_{\chi_{1L}^2}=0$. The second part ${\chi}_U^2{I}_{\chi_{1N}^2}$ presents the indeterminate part and ${I}_{\chi_{1N}^2}\epsilon \left[{I}_{\chi_{1L}^2},{I}_{\chi_{1U}^2}\right]$ is the measure of uncertainty.

Suppose now that the time to record *ith* neutrosophic count is *t*_*i*_. The test statistic, say ${\chi}_{2N}^2\epsilon \left[{\chi}_{2L}^2,{\chi}_{2U}^2\right]$ under neutrosophic statistics for this case is given by.

3 $${\chi}_{2N}^2=\sum_{i=1}^K\frac{{\left({N}_{iN}-{t}_i{\overline{R}}_N\right)}^2}{t_i{\overline{R}}_N};\kern0.5em {\chi}_{2N}^2\epsilon \left[{\chi}_{2L}^2,{\chi}_{2U}^2\right]$$

where ${\overline{R}}_N=\sum {N}_{iL}/\sum {t}_{iL}+\sum {N}_{iU}/\sum {t}_{iU}{I}_{{\overline{R}}_N};{I}_{{\overline{R}}_N}\epsilon \left[{I}_{{\overline{R}}_L},{I}_{{\overline{R}}_U}\right]$ and ${I}_{{\overline{R}}_N}\epsilon \left[{I}_{{\overline{R}}_L},{I}_{{\overline{R}}_U}\right]$ is a measure of indeterminacy. The neutrosophic form of test statistic ${\chi}_{2N}^2\epsilon \left[{\chi}_{2L}^2,{\chi}_{2U}^2\right]$ is expressed as follows.

4 $${\chi}_{2N}^2={\chi}_{2L}^2+{\chi}_{2U}^2{I}_{\chi_{2N}^2};{I}_{\chi_{2N}^2}\epsilon \left[{I}_{\chi_{2L}^2},{I}_{\chi_{2U}^2}\right]$$

Note that the proposed statistic ${\chi}_{2N}^2\epsilon \left[{\chi}_{2L}^2,{\chi}_{2U}^2\right]$ is the extension of the test statistic under classical statistics. The proposed test statistic ${\chi}_{2N}^2\epsilon \left[{\chi}_{2L}^2,{\chi}_{1U}^2\right]$ reduces to classical statistic ${\chi}_{2L}^2$ when ${I}_{\chi_{2L}^2}=0$. The second part ${\chi}_U^2{I}_{\chi_{2N}^2}$ presents the indeterminate part and ${I}_{\chi_{2N}^2}\epsilon \left[{I}_{\chi_{2L}^2},{I}_{\chi_{2U}^2}\right]$ is the measure of uncertainty. The proposed test will be implemented in the following steps

- **Step-1:** State the null *H*_0_ and alternative hypothesis *H*_1_.
- **Step-2:** State the level of significance *α* and decide about the critical region using the Chi-square table.
- **Step-3:** Reject *H*_0_ if ${\chi}_{1N}^2\epsilon \left[{\chi}_{1L}^2,{\chi}_{1U}^2\right]$ or ${\chi}_{2N}^2\epsilon \left[{\chi}_{2L}^2,{\chi}_{2U}^2\right]$ falls in the rejection area, otherwise accept *H*_1_.

## Conclusions

In this paper the Chi-square tests for K counts under neutrosophic statistics was presented. The test statistic of the proposed test when K counts were recorded at the same time and different times was proposed. The proposed test was the modified version of the existing test for K counts. The testing of the hypothesis procedure was explained with the help of a real example. From the pulse count data, it is concluded that the proposed test is effective to apply in uncertainty. In addition, the proposed test provides higher values of the power of the test. The proposed test guides the cardiologists to apply different treatment methods for patients. The proposed test using big data can be extended us future research.

## Data Availability

All data generated or analysed during this study are included in this published article.
